# HIV Care Outcomes Among Men Who Have Sex With Men With Diagnosed HIV Infection — United States, 2015

**DOI:** 10.15585/mmwr.mm6637a2

**Published:** 2017-09-22

**Authors:** Sonia Singh, Andrew Mitsch, Baohua Wu

**Affiliations:** 1 Division of HIV/AIDS Prevention, National Center for HIV/AIDS, Viral Hepatitis, STD, and TB Prevention, CDC.

Gay, bisexual, and other men who have sex with men (collectively referred to as MSM) represent approximately 2% of the U.S. population (*1*), yet in 2015, MSM accounted for 70% of all diagnoses of human immunodeficiency virus (HIV) infection, including 3% who also were persons who inject drugs (*2*). During 2008–2014, incidence of HIV infection decreased for groups in all transmission categories except MSM (*3*). Testing, linkage to and retention in care, and viral suppression are important in reducing HIV transmission. National HIV Surveillance System (NHSS)* data are used to monitor progress toward reaching national goals.^†^ To better guide prevention measures, CDC analyzed data from NHSS for MSM aged ≥13 years (excluding MSM who inject drugs) to determine stage at diagnosis of HIV infection and care outcomes. Among the 19,170 MSM with HIV infection diagnosed in 2015 in 38 jurisdictions with complete laboratory reporting, 3,666 (19.1%) had infection classified as stage 3 (acquired immunodeficiency syndrome [AIDS]) at diagnosis and 74.7% and 84.0% were linked to care within 1 month and 3 months, respectively. Among MSM living with diagnosed HIV infection at year-end 2014, 74.1% received any HIV care, 57.7% were retained in continuous care, and 61.2% had achieved viral suppression. Younger MSM and black or African American (black) MSM had the least favorable HIV care outcomes. Strengthening interventions that increase care and viral suppression among MSM, particularly those aged <25 years and black MSM with public and private partners is important.

All states, the District of Columbia, and U.S. territories report cases of HIV infection and associated demographic and clinical information to NHSS. CDC analyzed data for MSM aged ≥13 years (excluding MSM who inject drugs) reported through December 2016 from 38 jurisdictions (37 states and the District of Columbia)^§^ with complete laboratory reporting.^¶^ These jurisdictions accounted for 70.4% of MSM living with diagnosed HIV infection at year-end 2014 in the United States. Diagnoses of HIV infection are classified by severity of disease; stage 3 (AIDS) is the most severe. Stage 3 classification at the time of diagnosis and linkage to care were assessed among MSM living in any of the 38 jurisdictions at the time of diagnosis of HIV infection in 2015. Stage 3 classification at diagnosis of HIV infection was defined as having a CD4 lymphocyte count of <200/*μ*L, CD4 percentage of total lymphocytes of <14, or documentation of an AIDS-defining condition ≤3 months after a diagnosis of HIV infection. Linkage to care, defined as having documentation of ≥1 CD4 count or percentage or viral load (VL) tests, was assessed at ≤1 and ≤3 months after diagnosis of HIV infection. Receipt of care (any care and retention in care) and viral suppression were assessed among MSM with HIV infection diagnosed by December 31, 2013, and who were alive and resided (based on the most recent known address) in any of the 38 jurisdictions as of December 31, 2014 (i.e., persons living with diagnosed HIV infection). Any care (defined as having one or more CD4 or VL tests), retention in HIV care (defined as having two or more CD4 or VL tests ≥3 months apart), and viral suppression (defined as a VL of <200 copies/mL at most recent test) were assessed for 2014. HIV data routinely are statistically adjusted by using multiple imputation techniques to account for missing HIV transmission categories (*4*).

In 2015, in the 38 jurisdictions, 19,170 MSM received a diagnosis of HIV infection (Table 1). Blacks accounted for the largest number and percentage of HIV diagnoses (7,519; 39.2%) in this group. Overall, 3,666 (19.1%) of HIV infections diagnosed among MSM were classified as stage 3 at diagnosis. The percentage of HIV diagnoses classified as stage 3 increased with increasing age and was highest among whites (22.2%) and lowest among blacks (16.0%). The highest percentage of HIV infections diagnosed at an unknown stage was among blacks (25.9%) and lowest among whites (15.5%).

**TABLE 1:** Stage of disease at diagnosis of human immunodeficiency virus (HIV) infection, among men who have sex with men* aged ≥13 years, by age and race/ethnicity — National HIV Surveillance System, 38 jurisdictions,^†^ United States, 2015

Characteristic	No. (%)				
	Total HIV diagnoses in 2015	Stage 1 (CD4 ≥500 cells/*μ*L or ≥26%)	Stage 2 (CD4 200–499 cells/*μ*L or 14%–25%)	Stage 3 (AIDS) (OI or CD4 <200 cells/*μ*L or <14%)^§^	Stage unknown (No CD4 information)^¶^
**Age group at diagnosis (yrs)**					
13–19	**978 (5.1)**	273 (27.9)	404 (41.3)	60 (6.1)	242 (24.7)
20–24	**4,242 (22.1)**	1,234 (29.1)	1,680 (39.6)	360 (8.5)	968 (22.8)
25–34	**7,016 (36.6)**	1,897 (27.0)	2,487 (35.4)	1,115 (15.9)	1,518 (21.6)
35–44	**3,288 (17.2)**	817 (24.9)	978 (29.7)	906 (27.5)	587 (17.9)
45–54	**2,525 (13.2)**	554 (21.9)	713 (28.2)	818 (32.4)	440 (17.4)
≥55	**1,120 (5.8)**	223 (19.9)	307 (27.4)	407 (36.3)	184 (16.4)
Race/Ethnicity**					
Black/African American	**7,519 (39.2)**	1,757 (23.4)	2,613 (34.8)	1,203 (16.0)	1,946 (25.9)
Hispanic/Latino	**5,124 (26.7)**	1,289 (25.2)	1,831 (35.7)	1,033 (20.2)	971 (18.9)
White	**5,314 (27.7)**	1,666 (31.4)	1,644 (30.9)	1,178 (22.2)	826 (15.5)
Other^††^	**1,213 (6.3)**	286 (23.5)	480 (39.6)	252 (20.7)	196 (16.1)
**Total^§§^**	**19,170**	**4,998 (26.1)**	**6,568 (34.3)**	**3,666 (19.1)**	**3,938 (20.5)**

**Abbreviation:** AIDS = acquired immunodeficiency syndrome.

* Data statistically adjusted to account for missing transmission category.

^†^ The 38 jurisdictions were Alabama, Alaska, California, Colorado, Connecticut, Delaware, District of Columbia, Georgia, Hawaii, Illinois, Indiana, Iowa, Louisiana, Maine, Maryland, Massachusetts, Michigan, Minnesota, Mississippi, Missouri, Montana, Nebraska, New Hampshire, New Mexico, New York, North Dakota, Oregon, Rhode Island, South Carolina, South Dakota, Tennessee, Texas, Utah, Virginia, Washington, West Virginia, Wisconsin, and Wyoming.

^§^ Stage of disease at diagnosis of HIV infection based on first CD4 test performed or documentation of an AIDS-defining condition ≤3 months after a diagnosis of HIV infection.

^¶^ Includes persons with HIV disease classified as stage 0 (early infection, recognized by a negative HIV test within 6 months of HIV diagnosis: https://www.cdc.gov/mmwr/pdf/rr/rr6303.pdf).

** Black/African American, white, and other persons are non-Hispanic; Hispanic/Latino persons can be of any race.

^††^ Other race/ethnicity includes American Indians/Alaska Natives, Asians, Native Hawaiians/other Pacific Islanders and persons of multiple races.

^§§^ Because column totals for estimated numbers were calculated independently of the values for the subpopulations, the values in each column might not sum to the column total.

Among the 19,170 MSM with HIV infection diagnosed in 2015, 14,328 (74.7%) were linked to care within 1 month after diagnosis (Table 2). The percentage of MSM linked to care within 1 month after diagnosis was lowest among those aged 13–19 years (69.4%) and 20–24 years (70.1%) and highest among those aged ≥55 years (80.8%). The percentage of MSM linked to care within 1 month after diagnosis was lowest for blacks (69.3%) and highest for whites (81.1%). Overall, 16,112 (84.0%) MSM with HIV infection diagnosed in 2015 were linked to care within 3 months after HIV diagnosis. Percentages of MSM linked to care within 3 months after HIV diagnosis increased with increasing age, ranging from 81.0% among MSM aged 13–19 years to 87.6% among MSM aged ≥55 years. As was the case among MSM linked to care within 1 month of HIV diagnosis, the percentage of MSM linked to care within 3 months after HIV diagnosis was lowest for blacks (79.7%) and highest for whites (89.4%). Within each racial/ethnic group, linkage within 3 months varied little by age.

**TABLE 2:** Linkage to human immunodeficiency virus (HIV) medical care within 1 and 3 months of diagnosis of HIV infection, among men who have sex with men* aged ≥13 years, by race/ethnicity^†^ and age — National HIV Surveillance System, 38 jurisdictions,^§^ United States, 2015

Time to linkage to HIV medical care and age group at diagnosis (yrs)	Black/African American		Hispanic/Latino		White		Other^¶^		Total	
	No. HIV diagnoses	Linkage to care, No. (%)	No. HIV diagnoses	Linkage to care, No. (%)	No. HIV diagnoses	Linkage to care, No. (%)	No. HIV diagnoses	Linkage to care, No. (%)	No. HIV diagnoses	Linkage to care, No. (%)
Within 1 month of HIV diagnosis**										
13–19	618	410 (66.3)	205	155 (75.6)	104	80 (76.9)	51	34 (66.7)	**978**	**679 (69.4)**
20–24	2,222	1,470 (66.2)	1,073	775 (72.2)	687	528 (76.9)	260	200 (76.9)	**4,242**	**2,973 (70.1)**
25–34	2,845	1,982 (69.7)	1,971	1,465 (74.3)	1,734	1,382 (79.7)	467	370 (79.2)	**7,016**	**5,200 (74.1)**
35–44	915	665 (72.7)	1,068	826 (77.3)	1,083	883 (81.5)	222	183 (82.4)	**3,288**	**2,558 (77.8)**
45–54	632	470 (74.4)	614	476 (77.5)	1,117	936 (83.8)	161	133 (82.6)	**2,525**	**2,014 (79.8)**
≥55	287	213 (74.2)	192	148 (77.1)	590	501 (84.9)	52	42 (80.8)	**1,120**	**905 (80.8)**
**Total^††^**	**7,519**	**5,211 (69.3)**	**5,124**	**3,845 (75.0)**	**5,315**	**4,310 (81.1)**	**1,213**	**961 (79.2)**	**19,170**	**14,328 (74.7)**
**Within 3 months of HIV diagnosis^§§^**										
13–19	618	485 (78.5)	205	176 (85.9)	104	92 (88.5)	51	40 (78.4)	**978**	**792 (81.0)**
20–24	2,222	1,751 (78.8)	1,073	907 (84.5)	687	608 (88.5)	260	223 (85.8)	**4,242**	**3,489 (82.2)**
25–34	2,845	2,256 (79.3)	1,971	1,648 (83.6)	1,734	1,519 (87.6)	467	406 (86.9)	**7,016**	**5,829 (83.1)**
35–44	915	744 (81.3)	1,068	909 (85.1)	1,083	986 (91.0)	222	196 (88.3)	**3,288**	**2,835 (86.2)**
45–54	632	517 (81.8)	615	517 (84.1)	1,117	1,010 (90.4)	161	142 (88.2)	**2,525**	**2,186 (86.6)**
≥55	287	236 (82.2)	192	163 (84.9)	590	535 (90.7)	52	47 (90.4)	**1,120**	**981 (87.6)**
**Total^††^**	**7,519**	**5,989 (79.7)**	**5,124**	**4,319 (84.3)**	**5,315**	**4,751 (89.4)**	**1,213**	**1,053 (86.8)**	**19,170**	**16,112 (84.0)**

* Data statistically adjusted to account for missing transmission category.

^†^ Black/African American, white, and other persons are non-Hispanic; Hispanic/Latino persons can be of any race.

^§^ The 38 jurisdictions were Alabama, Alaska, California, Colorado, Connecticut, Delaware, District of Columbia, Georgia, Hawaii, Illinois, Indiana, Iowa, Louisiana, Maine, Maryland, Massachusetts, Michigan, Minnesota, Mississippi, Missouri, Montana, Nebraska, New Hampshire, New Mexico, New York, North Dakota, Oregon, Rhode Island, South Carolina, South Dakota, Tennessee, Texas, Utah, Virginia, Washington, West Virginia, Wisconsin, and Wyoming.

^¶^ Other race/ethnicity includes American Indians/Alaska Natives, Asians, Native Hawaiians/other Pacific Islanders and persons of multiple races.

** One or more CD4 or viral load test performed within 1 month after diagnosis of HIV infection during 2015.

^††^ Because column totals for estimated numbers were calculated independently of the values for the subpopulations, the values in each column might not sum to the column total.

^§§^ One or more CD4 or viral load test performed within 3 months after diagnosis of HIV infection during 2015.

Among 358,151 MSM living with diagnosed HIV infection at year-end 2014, a total of 265,280 (74.1%) received any care, 206,523 (57.7%) were retained in care, and 219,043 (61.2%) were virally suppressed (Table 3). The lowest percentages of retention in care (53.6%) and viral suppression (52.2%) were among black MSM. The highest percentages of receipt of any care (77.3%), retention in care (59.4%), and viral suppression (67.3%) occurred among whites. The percentage of MSM virally suppressed increased with increasing age for all racial/ethnic groups.

**TABLE 3:** Receipt of human immunodeficiency virus (HIV) care and viral suppression among men who have sex with men* aged ≥13 years, with diagnosis of HIV infection by December 31, 2013, who were alive on December 31, 2014, by race/ethnicity and age — National HIV Surveillance System, 38 jurisdictions,^†^ United States, 2014

Race/Ethnicity^§^ and age group (yrs) at year-end 2014	Total No.	Receipt of care, No. (%)		Viral suppression^†† ^No. (%)
		Any care^¶^	Retention in care**	
**All**				
13–19	1,292	1,000 (77.4)	755 (58.4)	663 (51.3)
20–24	15,777	11,761 (74.5)	8,569 (54.3)	8,020 (50.8)
25–34	62,569	45,723 (73.1)	34,027 (54.4)	34,507 (55.2)
35–44	79,169	57,919 (73.2)	44,384 (56.1)	47,327 (59.8)
45–54	121,470	91,096 (75.0)	71,705 (59.0)	77,554 (63.8)
≥55	77,875	57,781 (74.2)	47,082 (60.5)	50,972 (65.5)
**Total^§§^**	**358,151**	**265,280 (74.1)**	**206,523 (57.7)**	**219,043 (61.2)**
**Black/African American**				
13–19	773	579 (74.9)	424 (54.9)	360 (46.6)
20–24	9,381	6,781 (72.3)	4,813 (51.3)	4,246 (45.3)
25–34	27,792	19,614 (70.6)	14,171 (51.0)	13,379 (48.1)
35–44	24,205	17,047 (70.4)	12,877 (53.2)	12,712 (52.5)
45–54	31,274	22,215 (71.0)	17,472 (55.9)	17,378 (55.6)
≥55	16,437	11,354 (69.1)	9,098 (55.4)	9,315 (56.7)
**Total^§§^**	**109,863**	**77,590 (70.6)**	**58,854 (53.6)**	**57,389 (52.2)**
**Hispanic/Latino**				
13–19	316	263 (83.2)	200 (63.3)	191 (60.4)
20–24	3,242	2,504 (77.2)	1,916 (59.1)	1,891 (58.3)
25–34	16,715	12,054 (72.1)	9,416 (56.3)	9,637 (57.7)
35–44	22,581	15,780 (69.9)	12,846 (56.9)	13,419 (59.4)
45–54	24,927	17,898 (71.8)	15,008 (60.2)	15,703 (63.0)
≥55	11,364	7,908 (69.6)	6,870 (60.5)	7,186 (63.2)
**Total^§§^**	**79,146**	**56,407 (71.3)**	**46,256 (58.4)**	**48,027 (60.7)**
**White**				
13–19	115	84 (73.0)	67 (58.3)	61 (53.0)
20–24	2,107	1,645 (78.1)	1,210 (57.4)	1,279 (60.7)
25–34	13,797	10,684 (77.4)	7,907 (57.3)	8,850 (64.1)
35–44	26,550	20,508 (77.2)	15,097 (56.9)	17,404 (65.6)
45–54	58,134	45,149 (77.7)	34,533 (59.4)	39,492 (67.9)
≥55	46,177	35,401 (76.7)	28,489 (61.7)	31,731 (68.7)
**Total^§§^**	**146,881**	**113,471 (77.3)**	**87,303 (59.4)**	**98,815 (67.3)**
**Other^¶¶^**				
13–19	88	74 (84.1)	65 (73.9)	52 (59.1)
20–24	1,046	831 (79.4)	630 (60.2)	605 (57.8)
25–34	4,264	3,371 (79.1)	2,533 (59.4)	2,642 (62.0)
35–44	5,832	4,583 (78.6)	3,565 (61.1)	3,793 (65.0)
45–54	7,134	5,834 (81.8)	4,691 (65.8)	4,981 (69.8)
≥55	3,896	3,117 (80.0)	2,626 (67.4)	2,740 (70.3)
**Total^§§^**	**22,261**	**17,811 (80.0)**	**14,110 (63.4)**	**14,812 (66.5)**

* Data statistically adjusted to account for missing transmission category.

^†^ The 38 jurisdictions were Alabama, Alaska, California, Colorado, Connecticut, Delaware, District of Columbia, Georgia, Hawaii, Illinois, Indiana, Iowa, Louisiana, Maine, Maryland, Massachusetts, Michigan, Minnesota, Mississippi, Missouri, Montana, Nebraska, New Hampshire, New Mexico, New York, North Dakota, Oregon, Rhode Island, South Carolina, South Dakota, Tennessee, Texas, Utah, Virginia, Washington, West Virginia, Wisconsin, and Wyoming.

^§^ Black/African American, white, and other persons are non-Hispanic; Hispanic/Latino persons can be of any race.

^¶^ One or more CD4 or viral load tests performed during 2014.

** Two or more CD4 or viral load tests performed at least 3 months apart during 2014.

^††^ Viral load results of <200 copies/mL at the most recent viral load test during 2014. The cutoff value of <200 copies/mL was based on the U.S. Department of Health and Human Services recommended definition of virologic failure.

^§§^ Because column totals for estimated numbers were calculated independently of the values for the subpopulations, the values in each column might not sum to the column total.

^¶¶^ Other race/ethnicity includes American Indians/Alaska Natives, Asians, Native Hawaiians/other Pacific Islanders and persons of multiple races.

## Discussion

Among MSM aged ≥13 years with HIV infection diagnosed in 2015, 19.1% of infections were classified as stage 3 at the time of diagnosis. This suggests that one in five MSM have advanced immunosuppression at the time of diagnosis, highlighting the urgent need for screening. The percentages of MSM linked to care within 1 month and 3 months after diagnosis of HIV infection, were 74.7% and 84.0%, respectively. Among MSM living with HIV diagnoses at year-end 2014, 57.7% were retained in care and 61.2% had achieved viral suppression; these percentages fall short of the national goals for persons living with HIV infection of 85% linkage to care within 1 month after HIV diagnosis, 90% retention in care, and 80% viral suppression (*5*). HIV testing, linkage to and engagement in care, and achieving viral suppression are important to prevent disease progression and reduce further transmission of HIV infections.

The percentage of HIV diagnoses classified as stage 3 at the time of diagnosis among MSM increased with increasing age. Because the natural course of untreated HIV infection results in severe immunosuppression several years after the time of infection, younger patients are less likely than are older patients to have developed severe immunosuppression by the time of diagnosis. The low percentage (16.0%) of HIV diagnoses classified as stage 3 among black MSM suggests that, compared with other racial/ethnic groups, blacks might receive testing sooner after infection, leading to a lower percentage of infections classified as stage 3 at the time of HIV diagnosis.

Percentages of linkage to care and viral suppression were lowest among younger MSM, and all care and treatment outcomes were least favorable for black MSM. Compared with 2010 findings based on data from 19 jurisdictions (*6*), HIV care outcomes in 2015 have improved for MSM, including linkage to care (77.5% in 2010 compared with 84.0% in 2015), which was assessed at 3 months after HIV diagnosis, as well as retention in care (50.9% compared with 57.7%), and viral suppression (42.0% compared with 61.2%). Although these advances in HIV care outcomes are promising, 52.0% of young MSM with HIV infection do not know that they are infected (*3*). Persons who become aware of their HIV infection are more likely to reduce risk behaviors and can begin HIV medical care and treatment (*7*). CDC recommends routine voluntary HIV screening for all persons aged 13–64 years and annual testing for persons at high risk for HIV infection; sexually active MSM might benefit from more frequent screening (i.e., every 3–6 months) (*8*). Testing is the gateway to the continuum of care for persons who test positive and, along with risk assessment, the gateway to preexposure prophylaxis for those who test negative. To prevent HIV infection among MSM, care outcomes can improve by increasing access and adherence to antiretroviral therapy by persons already infected and to preexposure prophylaxis by those not known to be infected (*9*).

The findings in this report are subject to at least five limitations. First, analyses were limited to 38 jurisdictions with complete reporting of all levels of CD4 and VL test results; these jurisdictions might not be representative of all MSM living with diagnosed HIV infection in the United States. The included jurisdictions accounted for 70.4% of MSM living with diagnosed HIV infection at year-end 2014. Second, overall national data might not be applicable to all states. Third, some cases of HIV infection are reported to CDC without an identified risk factor. Statistical adjustments were applied for missing risk factor information; however, misclassification might have occurred (*4*). Fourth, the most recent VL might not be indicative of consistent viral suppression. Finally, some diagnoses of HIV infection are reported without CD4 data, and in these cases, stage of disease at HIV diagnosis cannot be determined; therefore, comparisons of stage of disease by age and race/ethnicity should be interpreted with caution.

MSM accounted for the majority of diagnoses of HIV infection made in 2015 and the majority of persons living with HIV at year-end 2014. Addressing HIV infection among MSM and the ongoing racial/ethnic disparities in HIV care outcomes among MSM is important to reduce HIV infections in the United States. CDC is pursuing a high-impact prevention approach (*10*) to reduce the number of HIV infections and to increase the effectiveness of HIV prevention and care activities through partnerships with federal, state, and local health agencies and their public and private sector partners. CDC currently funds prevention, surveillance, research, and evaluation programs for MSM, including racial/ethnic minority MSM.** To further reduce HIV transmission among MSM, targeted HIV testing and strengthened measures to increase linkage to care, retention in care and achievement of viral suppression are important, particularly for MSM aged <25 years and black MSM.

**Summary**

**What is already known about this topic?**

Gay, bisexual, and other men who have sex with men (collectively referred to as MSM) represent approximately 2% of the U.S. population, yet in 2015 MSM accounted for 70% of all diagnoses of human immunodeficiency virus (HIV) infection, including 3% who also were persons who inject drugs. National goals for persons living with HIV infection include linkage to care for 85% within 1 month of diagnosis, retention in care for 90%, and viral load suppression for 80% by 2020.

**What is added by this report?**

In 2015, 19% of HIV infections diagnosed among MSM were classified as stage 3 (acquired immunodeficiency syndrome), and 75% of MSM with diagnoses of HIV infection were linked to care within 1 month. MSM who were black or African American and MSM aged <25 years were less likely to be linked to care within 1 month of diagnosis of HIV infection compared with other racial/ethnic and age groups. Among MSM living with diagnosed HIV infection at year-end 2014, 74% received any care, 58% were retained in care, and 61% had achieved viral suppression. Retention in care and viral suppression were low in all MSM, particularly black or African American MSM.

**What are the implications for public health practice?**

Tailored strategies for MSM that increase care and achieve viral suppression, particularly among young MSM and black or African American MSM, are needed to reduce HIV infections, improve health outcomes for persons living with HIV infection, and reduce HIV-related health disparities.

## Footnotes

* The National HIV Surveillance System is the primary source for monitoring human immunodeficiency virus (HIV) trends in the United States. Through the system, information about cases of HIV infection is collected, analyzed, and disseminated.

† The national goals to be accomplished by 2020 are as follows: 1) 85% of all persons with newly diagnosed HIV infection to be linked to care within 1 month after HIV diagnosis, 2) 90% of persons living with diagnosed HIV infection to be retained in care, and 3) 80% of persons living with diagnosed HIV infection to have a suppressed viral load.

§ The 38 jurisdictions were Alabama, Alaska, California, Colorado, Connecticut, Delaware, District of Columbia, Georgia, Hawaii, Illinois, Indiana, Iowa, Louisiana, Maine, Maryland, Massachusetts, Michigan, Minnesota, Mississippi, Missouri, Montana, Nebraska, New Hampshire, New Mexico, New York, North Dakota, Oregon, Rhode Island, South Carolina, South Dakota, Tennessee, Texas, Utah, Virginia, Washington, West Virginia, Wisconsin, and Wyoming.

¶ The criteria for complete reporting were as follows: 1) The jurisdiction's laws or regulations required the reporting of all CD4 and viral load results to the state or local health department, 2) laboratories that perform HIV-related testing for the jurisdictions had reported a minimum of 95% of HIV-related test results to the state or local health department, and 3) by December 31, 2016, the jurisdiction had reported (to CDC) at least 95% of all CD4 and viral load test results received from January 2014 through September 2016.

** https://www.cdc.gov/msmhealth/msm-programs.htm.
